# Colombian forensic genetics as a form of public science: The role of race, nation and common sense in the stabilization of DNA populations

**DOI:** 10.1177/0306312715574158

**Published:** 2015-12

**Authors:** Ernesto Schwartz-Marín, Peter Wade, Arely Cruz-Santiago, Roosbelinda Cárdenas

**Affiliations:** Department of Anthropology, Durham University, Durham, UK; Social Anthropology, School of Social Sciences, The University of Manchester, Manchester, UK; Department of Geography, Durham University, Durham, UK; School of Critical Social Inquiry, Hampshire College, Amherst, MA, USA

**Keywords:** Colombia, common sense, forensic genetics, nation, racialization

## Abstract

This article examines the role that vernacular notions of racialized-regional difference play in the constitution and stabilization of DNA populations in Colombian forensic science, in what we frame as a process of public science. In public science, the imaginations of the scientific world and common-sense public knowledge are integral to the production and circulation of science itself. We explore the origins and circulation of a scientific object – ‘La Tabla’, published in Paredes et al. and used in genetic forensic identification procedures – among genetic research institutes, forensic genetics laboratories and courtrooms in Bogotá. We unveil the double life of this central object of forensic genetics. On the one hand, La Tabla enjoys an indisputable public place in the processing of forensic genetic evidence in Colombia (paternity cases, identification of bodies, etc.). On the other hand, the relations it establishes between ‘race’, geography and genetics are questioned among population geneticists in Colombia. Although forensic technicians are aware of the disputes among population geneticists, they use and endorse the relations established between genetics, ‘race’ and geography because these fit with common-sense notions of visible bodily difference and the regionalization of race in the Colombian nation.

## Introduction

Technologies that use DNA to identify human remains or link a suspect and a crime work by drawing mathematical associations between an individual person, a DNA sample, and the DNA profile of a relatively fixed ‘reference population’. DNA ‘fingerprints’, known as ‘profiles’ in Colombia, intertwine an individual with a population; that is, a DNA fingerprint needs a population to gain its meaning (M’charek, 2000). As one forensic anthropologist interviewed for this article explained, ‘Without populations there is no way to produce certainty’.

This article explores how the unwieldy and unruly construction of ‘population’ fits common-sense notions of race, nation and region in Colombia. In Colombia, a table describing allelic frequencies for four populations defined as distinctive (Paredes et al., 2003), commonly known among forensic technicians as *La Tabla* (The Table), is widely used for forensic purposes. It fulfils a normative role in forensic science by providing the genetic reference populations with which DNA matching is carried out. La Tabla establishes certain relations between populations, regions, race and genetics, yet the populations it constructs are far from being comfortably settled among population geneticists; indeed, they are questioned and criticized. Expert criticisms of La Tabla are not directed towards its usefulness – it has been a standard tool for forensic genetics in Colombia for over ten  years. Rather, they question the scientific validity of the argument that La Tabla presents populations.

Matching the DNA found in a sample to an individual is not as straightforward as comparing fingerprints or dental records. Because it would be an impossibly cumbersome task to compare the genomes in their entirety, specific genetic traits – short tandem repeats (STRs, usually called ‘alleles’ in the laboratory)^1^ – are analysed to determine the probability that they occur in another random individual *in the population to which the individual is deemed to belong* (Amorim, 2012: 261–263). This is expressed as a likelihood ratio (LR) or a random match probability. Forensic genetics, despite the gloss given by practitioners and the mass media, is messy and fluid. The DNA populations at the centre of its probabilistic logics are unruly (Gannett, 2003) and entangled with racialized narratives of sameness and difference.

In forensic practice – for example, in Colombia’s National Institute of Legal Medicine and Forensic Science (Instituto Nacional de Medicina Legal y Ciencias Forenses (INMLyCF)) – technicians known as *peritos* (experts) routinely use La Tabla’s reference populations to calculate match probabilities after having found a preliminary match between suspect and reference sample. The peritos are aware of the debates about La Tabla, but use it nonetheless. It works for pragmatic purposes, and the links it establishes between race, region and population appear, at one level, obvious, commonsensical and unproblematic. However, La Tabla’s explicit use of race can be uncomfortable for the peritos, a discomfort they partly resolve by emphasizing what they describe as the ‘bioethical’ nature of the DNA markers used to make matches. Compared to technologies such as fingerprints, which were actively divested of the racialized narratives built around them throughout the 19th and 20th centuries (Cole, 2002: 101–103), forensic DNA markers are seen as inherently independent of phenotypical associations with race, thus avoiding accusations about racial prejudice. However, forensic practice incorporates racialized narratives and logics in the use of reference populations. Inside courtrooms where the peritos’ expert reports circulate among attorneys, judges, *fiscales* (public prosecutors), criminal investigators and the relatives of victims, La Tabla’s DNA racialized reference populations tend to disappear almost entirely, leaving only the probabilities that are said to match an individual with a sample and make a genetic profile into a ‘unique barcode’ and thus the ‘gold standard’ in forensic identification.

We explore the multiple lives of this scientific object – La Tabla – as it moves through the worlds of the population geneticists, the peritos and the courtrooms. Despite what some population geneticists see as scientific flaws, La Tabla has become a national standard in forensic genetic practice. To explain the successful standardization of a disputed scientific object, we explore how common-sense notions of national and racial difference are accommodated into scientific knowledge and become integral to its successful circulation. We argue that this can be best understood as a case of what Wynne (2005) has defined as public science, in which

[i]maginations of the public world, however that is construed, can be taken as integral to scientific knowledge-generation, not simply as afterthoughts. Thus I talk of *public* science, in the sense of scientific knowledge in which we may identify such implicit human–public dimensions as part of the science itself. (p. 68)

Based on ethnographic work in the Colombian forensic system, we show how the coexistence of multiple and potentially conflicting constructions of difference is facilitated by sometimes separating and sometimes conflating the visible (race, ethnicity, region) and the invisible (the DNA). Depending on the context in which forensic genetics is working, different scientific objects (facts) and narratives (fictions) about race and nation are mobilized, making genetic populations appear, disappear and sometimes become problematized.

The imaginaries and practices of sameness/difference pertaining to race, nation and region enter into the production of science through the making and circulation of forensic DNA populations. This dovetails with M’charek’s (2013) practice-centred approach that argues race is simultaneously factual and fictional. Our focus on everyday scientific practices allows us to go beyond the accounts given by scientists about the meaning of their own work and instead follow how a scientific object unfolds in different social arenas (Clarke, 2005). Scientists and other stakeholders find themselves mobilizing existing narratives as ‘fictions’ or ‘facts’ – such as the notion that Colombia is a country of ‘natural regions’ with corresponding genetic populations – to produce coherence or to challenge truth claims.

Seeing the intertwining of facts and fictions helps explain how novel sets of populations and political goals emerge hand in hand. For Colombian forensic genetics, notions of racial difference coexist with narratives of *mestizaje* (cultural and racial mixture) that supposedly make such differences inapplicable in the first place. Indeed, the tensions between notions of genetics, mestizaje, race and regional difference are found throughout the forensic identification process, including post-mortem forms that ask for the race (*raza*) of the deceased and the framing of DNA profiles as based on ‘bioethical markers’ that avoid links with physical appearance. Forensic specialists have developed many strategies to deal with the tensions arising from the conflicting mobilization of racial, ethnic and national categories in the process of forensic identification. Racial difference both appears and disappears in Colombian forensic practice, as it does in Colombian public life (Wade et al., 2014).

In the context of Colombia, redefined as multicultural and pluriethnic in its 1991 constitutional reform, which also gave special rights to Afro-Colombian and indigenous communities, the reiteration of racialized-regional difference, via genetics, takes on a slightly different meaning. While racial–regional diversity has long been recognized, the mestizo Andean highlands were dominant and defined the essence of the nation, while the Black and indigenous regions were more marginal. With the 1991 reform, this weighting was, in theory, re-balanced by the explicit recognition of difference, which also opened the possibility of debating racial inequality. In practice, the tension between sameness – ‘we are all a *mescolanza* [a mixture]’ – and difference – ‘we are not the same’ – is rehearsed again, and race both appears – in the recognition of ‘Black communities’ and talk of racism – and disappears – in assertions of tolerant multiculturalism and increasing mixture.

There are various studies of the public dimensions of forensic science (Jasanoff, 1998; Jordan and Lynch, 1998; Lynch and Jasanoff, 1998; Lynch et al., 2008). A rich literature also explores race, nation and genetics (e.g. Fujimura and Rajagopalan, 2011; Nash, 2013; Schramm et al., 2012; Wade et al., 2014; Wailoo et al., 2012). Within this latter literature, a subset of studies focuses on the relation between forensic genetics and notions of racial, ethnic and national difference (e.g. Duster, 2006; Kahn, 2012; M’charek, 2000, 2008, 2013; M’charek et al., 2012, 2014; Skinner, 2013). However, little research has been done on scenarios outside European or US contexts (Heinemann et al., 2012). Kahn (2012) accounts for the continuing traces of forensic racial classification in US legal practice in terms of the ‘inertial power’ of race in US society; we show that, in Colombia, racialized ideas have a certain inertia, inscribed in images of regional difference, that take on a genetic dimension. But such ideas are also constantly contested by concepts of DNA as too complex to be reduced to race and as having the status of a ‘truth machine’ (Lynch et al., 2008), which gives it the power to rise above race.

First, we present a brief history of forensic genetics in Colombia, as it responded to violent events in the 1980s and 1990s, before its standardization in the early 2000s. The second section examines some of the disputes that population geneticists have with La Tabla. Third, we contrast those disputes with the everyday explanations by peritos of the logic behind producing four genetic populations and its links to racialized imaginaries of the Colombian nation. Finally, we explore the work of forensic genetics in the Colombian courtrooms, as seen by relatives of the disappeared, public prosecutors and police forces.

The data for this article come from 6 months of ethnography at Colombia’s National Institute of Legal Medicine and Forensic Science (INMLyCF). Our ethnography included observations in courtrooms, five focus groups with police officers preparing to become criminal investigators and three group interviews and many personal interviews with the forensic peritos (technical experts) of the INMLyCF and the Fiscalía Nacional or Public Prosecutor’s Office (with 26 participants in total). We also did 25 semi-structured interviews with population geneticists in various research organizations in Bogotá and with users of forensic science, mostly public prosecutors and relatives of victims of forced disappearance.

## A brief history of forensic genetics in Colombia

The INMLyCF and the other laboratories of the National System of Legal Medicine should have proofs based on the application of scientific knowledge to our national reality, which is why it is of vital importance – for forensic genetics – to know the genetic composition of our country’s population, so we can generate a correct interpretation of results, this in turn will allow the judicial apparatus to make an approximation to truth, and in this way contribute with the correct application of justice. (Terreros, 2010)

At the end of the 1960s, the first paternity cases were solved by Emilio Yunis, who is now recognized as a pioneer of human genetics in Colombia, at the Colombian Institute of Family Welfare (Instituto Colombiano de Bienestar Familiar, ICBF). The standardization of blood groups to solve paternity cases began in 1968 as a result of Law 75, designed to protect minors and guarantee their right to know their biological origins. In those days, paternity cases were solved by an ‘anthropological–hereditary–biological’ examination, based on the analysis of blood groups, as well as ‘pathological, morphological, physiological and intellectual characters transmissible between generations’ (Article 7, Law 75 of 1968, in Guerrero Diaz, 2010).

The move from the anthropological–hereditary–biological examination to the widespread use of genetic technologies as a ‘gold standard’ took several decades. Before the move, the reliability and impartiality of forensic science, including genetics, were often publicly questioned, especially when related to high-profile events of violence. This period of Colombia’s history was especially violent, and impunity and corruption reigned. In the 1980s and beginning of the 1990s, when the war against drug dealers and armed insurgents was at its height, forensic genetics was increasingly being used in the identification of human remains (Rodriguez, 2004). At the same time, the use of physical anthropological criteria of sex, age, build and ‘racial ancestor’ (*ancestro racial*),^2^ in combination with facial reconstruction, continued to be used. Genetic identification technologies at the time consisted of a ‘curious combination of blood groups and DNA markers, which did not yield a very high likelihood ratio or random match probability’ (Manuel Paredes, 2011, interview).

Iconic events in which forensic science failed to provide robust evidence became milestones of change, challenging impunity and lack of due forensic process (Pardo, 2000; Rodriguez, 2004). A famous example is the case of ‘Operación Cirirí’ (Lalinde, 2008). Mrs Lalinde, the founder of the Operación Cirirí movement, is the mother of Luis Fernando Lalinde, a disappeared combatant extra-officially executed by the Colombian armed forces in 1984. She was the first layperson who, with the help of forensic anthropologists and 12 years of arduous labour, during which she was threatened and illegally incarcerated, overturned the expert opinion of the geneticist who had carried out the DNA testing for the case, Emilio Yunis. Yunis had claimed that ‘the remains examined with DNA techniques do not correspond to offspring of Mrs Lalinde … and these results are sufficient, irrefutable and unmodifiable’ (Pardo, 2000). In response to Yunis’ report, the Lalinde family asked for and finally received two international and independent identification reports stating that the remains examined did belong to Mrs Lalinde’s offspring (Rodriguez, 2004).

The Lalinde case was a public blow to the existing forensic system and its identification practices. In response to such challenges to forensic credibility, in 1993 a genetic forensics laboratory was created at the INMLyCF, which, around 2000, established Colombia’s first DNA database following Combined DNA Index System (CODIS) protocols. CODIS is a DNA database and software platform established by the Federal Bureau of Investigation (FBI), in which genetic profiles from individuals convicted of felonies or looking for their relatives are stored; it is based on thirteen STRs (alleles) that are standardized for purposes of comparability. Similar CODIS-like databases and collection protocols exist worldwide (Wagner and Katsanis, 2012). In Colombia, DNA profiles of unidentified human remains and relatives of the disappeared are included in the INMLyCF’s database, which has a total of about 19,000 profiles.

The standard set of reference populations known as La Tabla was used for the first time in 2003. La Tabla became standardized into the INMLyCF’s database and its internal software. It also defined the standard reference populations used by almost all state and privately funded laboratories in Colombia because it was adopted by the Colombian Institute of Family Welfare for paternity testing; anyone wanting to get a share of the profitable paternity testing market is obliged to use La Tabla.^3^ As a consequence, La Tabla and the populations it contains have become an obligatory point of passage (Latour, 1987) for all forensic genetic enquiries in the thousands of civil and penal cases that are processed by the courts each year.^4^

**Figure 1.**
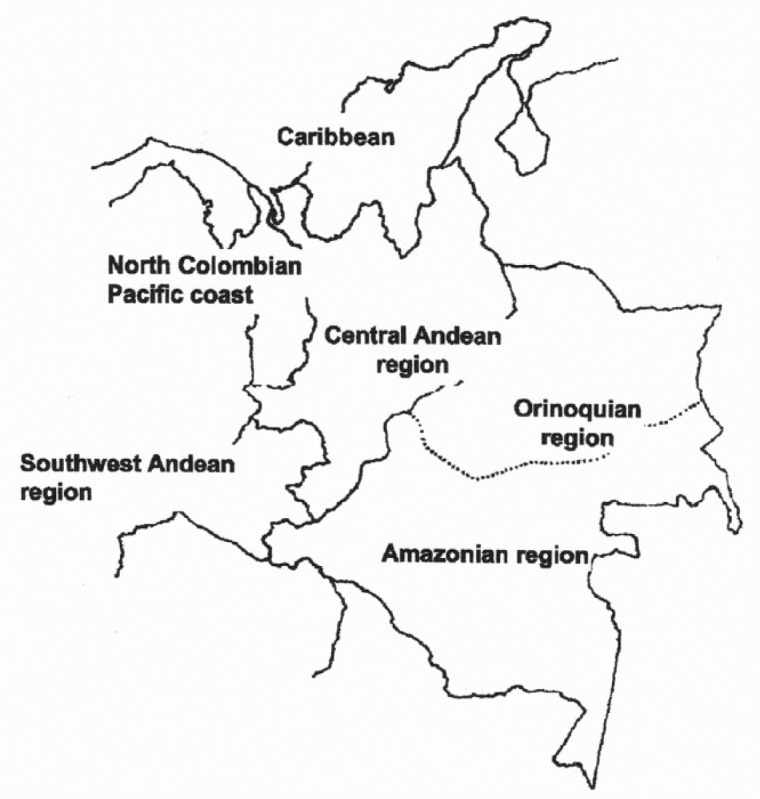
Map from Paredes et al. (2003), showing the regions described (with permission from Elsevier).

According to one of La Tabla’s authors, Manuel Paredes, Colombian geneticists thought at the time that obliging them to use standard national reference populations was unnecessary because genetic studies of different regions of Colombia already existed. Emilio Yunis, for example, had been developing population databases for ‘Caucasian-Mestizo’, ‘Black’ and Amerindian populations (Yunis and Yunis, 1999). According to La Tabla, there are four main population groups, corresponding to specific regions of the country. These population groups are described in overtly racialized terms: The ‘North Colombian Pacific coast’ has an ‘African-descendant’ population, as does the ‘Caribbean’ region; the ‘Central Andean region’ that includes the Amazonian and Orinoquian regions have ‘Mestizos’ populations, while the populations of the ‘Southwest Andean region’ have an ‘important Amerindian component’ (Paredes et al., 2003: 68). The geneticists used ‘historical documentation’ (which appears to be three texts written by historians and economists) to define the four basic regions, which were then used to organize the genetic data drawn from 1429 individuals during paternity testing: ‘The use of clustering methods (UPGMA) showed a complete correlation of the genetic data with the historical classification’ (Paredes et al., 2003: 68) (Figure 1).

## La Tabla among population geneticists

The data presented in La Tabla lie at the centre of almost all forensic work that deals with genetic evidence. The journal article containing La Tabla is six pages long and contains four tables reporting different frequencies of the thirteen STRs used as standard genetic markers in the CODIS database. The authors claim that they found ‘a complete correlation’ between the clustering of DNA data and the regions derived from historical sources, but this argument has been criticized on at least two counts. First, some geneticists contend that there are no significant genetic differences between the four populations described in the article. Second, critics argue that genetic populations cannot be described in a robust manner by technologies employing the thirteen STRs.

### No significant differences?

In relation to the first criticism, even one of the authors of the article containing La Tabla voiced scepticism about the significance of the genetic differences among the populations described in this article:

Well, you should know that we were very surprised by the results of our study, we thought that we would find clear differences at least between the coast and the interior, but we did not … to be honest, none of us expected those results. (Interview, 2012)

For this co-author, La Tabla was supposed to constitute a first phase of forensic research on Colombian genetic diversity, followed by a more rigorous exercise – but this second phase never happened. As we will see below, this criticism is also implicit in the experience of the peritos who use La Tabla and find that it makes little difference which of the four tables they use when generating a probability ratio for a match.

The ‘complete correlation’ found between the clustering of the genetic data and the historical classification was made without any reference to F_st_ (fixation index) values – measures of genetic distance – which are often used in this kind of analysis (e.g. Rubi-Castellanos et al., 2009). Genetic distance provides geneticists with the mathematical foundation to assess whether they have found substructure or differentiation within a population or differentiation between populations. In the language of modern genetic science, if you don’t have F_st_ values, you can’t assess how much genetic differentiation there is. And La Tabla did not present any F_st_ data or refer to genetic distances between its four population groups.

We argue that common-sense imaginations of Colombia as a ‘country of regions’ played a significant role in the way La Tabla was produced and how it managed to achieve its pragmatic dominance as a tool used routinely by forensic technicians. In the public imaginaries of the Colombian nation, the country is generally seen as comprising four or five distinct regions – sometimes called natural regions – divided by deeply rooted histories, weaving together geographical, climatic and cultural differences (Centro de Investigación y Educación Popular, 1998; Instituto Geográfico Agustín Codazzi (IGAC), 2002; Zambrano and Bernard, 1993). Although talking about regions is not *ipso facto* talking about race,

history gave race a regional structure, such that race cannot be simply understood as a social construction around biology, but must also be seen as a social construction around region. In Colombia it is impossible to consider race without looking at region. (Wade, 1991:44)

Racial difference thus constitutes a fundamental aspect of regional difference, especially in relation to blackness, which is very powerfully associated with the Pacific coastal region and, to a lesser extent, with the Caribbean littoral (Wade, 1993).

The populations constructed in La Tabla are an example of this grammar of racialized regions, which is deeply embedded into forensic practice: These populations are written into SIFMELCO, the on-line platform and software used to administer the DNA database in the INMLyCF. The use of racialized categories in forensic DNA matching protocols is not only a Colombian feature. When calculating LRs for DNA matches, US forensic technicians also continue to use reference populations classified by the US census race categories (Butler et al., 2003; Kahn, 2012), and this precedent may well have been an additional factor influencing the production of La Tabla.

Although Paredes has stated (interview, 2012) that La Tabla shows only minute differences between populations, he maintains that the differences are due to the fact that people tend to stay in the same region for various generations; the article states that 80 percent of the sampled individuals were born in the ‘locality’ where they were sampled and approximately 63 percent lived in the same ‘geographic region’ as his or her parents, although neither ‘locality’ nor ‘region’ is defined (Paredes et al., 2003: 67). In light of the major civil conflicts that Colombia has faced since late nineteenth century, the doubling, between 1938 and 1964, of the proportion of the population living in urban areas, and the 4.9 million people estimated to have been *desplazados* (internally displaced) between 1985 and 2009, establishing links between genetic populations and geographic demarcations is, in the eyes of many population geneticists in Colombia, a complex and even impossible task.

### Inadequate tools to identify difference?

Jaime Bernal and Emilio Yunis, senior geneticists in Colombia, share the first criticism that the divisions this article makes are not supported by its data and that forensic practice can do as well with or without the four different populations, as the data presented do not establish significant genetic differences between them. The fact that the genetic populations in La Tabla more or less coincide with common-sense notions of Colombia’s regions and populations suggests the imposition of pre-existing frames on the data. But Bernal and Yunis add a second critique, which is that the thirteen CODIS-based STRs are not an adequate tool to distinguish between regions or populations. This second criticism of La Tabla derives from geneticists’ claims that its methods are not fit for the purpose of describing genetic populations. For example, STR markers are highly polymorphic (i.e. highly variable), which allows them to overcome the kind of genetic differences commonly attributed to ethno-racial and bio-geographical groupings on the basis of other kinds of markers (e.g. AIMs or ‘ancestry informative markers’). This does not mean that Bernal and Yunis reject the idea of a regionalized nation. For Yunis, Colombia is an ‘ethnic mosaic’, in part because of ‘cultural mestizaje’ (interview, 2012), but also because it is a country of regions in which both race and genes are regionalized, resulting in a ‘black Colombia’, represented mainly by the coastal regions, a ‘Mestizo Colombia’, with a strong ‘Caucasoid’ component, strongest in the Andean interior, and with indigenous components most evident in the far southwest (Yunis, 2006, 2009: 19, 361–365). As discussed above, Yunis developed DNA databases in the 1990s for Caucasian-Mestizos, Black and Amerindian populations. But he criticizes La Tabla:

I think that using STRs to differentiate populations at the local level [is wrong] – this is a global technology, using it to describe local populations means that you do not understand the first thing about existing forensic technologies! With thirteen STRs you need something like 700 billion individuals to find the same genetic profile, and with twenty markers even more; so using these markers to state what we already know does not make a lot of sense. (Paraphrase from interview, 2012)

For Yunis, STRs are not fit for the purpose of bringing any detail to what is already known. For Bernal, it is not necessary to carry out a genetic study to say roughly that the coastal populations have ‘African genes’ (interview, 2012). In Bernal’s view, marked by the anti-reductionist and humanist tradition of the Institute of Human Genetics at the Pontificia Javeriana University, which he directs (see Restrepo et al. 2014), it is more than a question of which genetic markers to choose. For him, using genetic data without a careful consideration of demographic, historical and cultural aspects is like trying ‘to write a cheque with the leg of a table’ (interview, 2012).

Diogenes (pseudonym) is a population geneticist who has deeply and publicly questioned the making of such geographical-genetic clusters.^5^ His own studies reveal a rather less neat relation between common-sense national imaginaries and their genetic representations.^6^ For Diogenes, it is not only that the four populations of La Tabla are redundant – they state without appropriate data what ‘everybody knows’ about Colombia – but also that using existing racialized categories to capture human diversity in Colombia might not be adequate in the first place. He argues that population categories and their relation to geography should be based on a detailed study of each locality’s history, demography and genealogical knowledge:^7^

For me it is more adequate to use local categories based on interviews, self-ascription to a group or a community, demography, matting patterns and the number of generations that the families have lived in the area. That, I think, is far better than to say that they are Afro-Colombians, Caribbeans, Mestizos or Caucasoid-Mestizos … I think these categories are too broad and too ambiguous, and they are not really appropriate because these categories don’t recover much of the history of the populations. These categories can, and should be adjusted. This is done when you go there and find out for yourself the history of the population. (Interview, 2012)

La Tabla was compiled through ‘the numerous paternity cases at hand, which provided good biological material with existing informed consent and came from all the corners of the Colombian territory’ (Paredes, 2012, interview). For Diogenes, the technical need to fill a table with allelic frequencies, characteristic of many forensic geneticists’ practice, is exactly the opposite of the proper practice of population genetics research:

Diogenes: Of course it is not the same to take one hundred random samples from the freezer – using your paternity cases – type them and then move on to say that you represent the genetic population of this or that place: like Antioquia [a Central Andean sub-region], the Caribbean or whatever. Here in Colombia almost anyone can claim to be doing population genetics, and I say no […] The characterization of Colombia that I am now doing will take like eight or ten years. Just so you can imagine, for four departments I have 1100 samples with genealogies and everything.

E.S.M.: So do you think a map done at a population level with demography and all the rest, against this one, let’s say done with the forensic samples at hand, will really change?

Diogenes: Yes of course! Definitely. Radically! … Radically! For example, we right now … in the work we did for El Tolima and Huila [departments of Colombia], we have not found a geographical relationship with anything, but we have had populations, for example the [indigenous] Wayúu, which are linked to the geographical zone in which they live. Of course both populations have a very different history … so the genetic-geographic relation is not necessarily true.

This is a critique not only of Paredes but more generally of the common-sense grammars of difference that Colombian population geneticists have used for so long (Restrepo et al., 2014), and which Diogenes considers an impediment for accurately describing the genetic diversity of Colombia. Paredes responds to these criticisms by remembering when geneticists criticized the idea of using standard reference populations to produce forensic reports:

Geneticists claimed that the INMLyCF was obstructing the development of the field of forensic genetics with its stubbornness in asking for a reference population to provide results, and then again just amongst geneticists, the idea that there were four subpopulations was questioned, something that I don’t think is the case anymore … however yes, they questioned these different populations. But then again, they use the tables based on our large databanks to do their work, because despite all these criticisms they [the tables] are rigorous and well done, and we have not had any complaints about their efficacy. (Paredes in closing fieldwork conference/debate with research participants of the INMLyCF, 22 June 2012)

For Paredes, as for many other forensic geneticists dealing with the everyday workload of an overwhelmed forensic system, the question of the artificiality of the population categories is solved by the fact that La Tabla works and that his critics themselves constantly work with it.

The critics prefer an approach that envisages new and different categories for population genetics, and they point to the lack of robust evidence for distinguishing four different populations or criticize the use of STR markers. However, common-sense ideas of the Colombian nation and its regions infuse the DNA populations of La Tabla and are taken for granted by many of its critics, who also reproduce the idea of a basic correlation between region, race and genetics.

## La Tabla and race among forensic genetic technicians

What better than an article that – for better or worse – everybody talks about … and time goes by and it survives … in that sense the paper [Paredes et al.] has excelled in a test that goes way beyond my criticisms. In the end they say: ‘criticize all you want, we use it just the same all over the country … and everybody knows it, and we have used it for over a decade’ […] Historically he [Paredes] has no data to sustain those classifications and we have still used it for ten years, nonetheless!! (Diogenes, 2012, interview)

The exigencies of standardization – especially its needs for stability, fixedness and reproducibility – make it difficult for peritos to engage in a systematic way with the debates going on among population geneticists about the artificiality or appropriateness of existing categories to classify human diversity. They are certainly aware of these debates – after all, almost all of them had studied (or were still studying) in a genetics research institute in Bogotá or had attended conferences in which population geneticists challenged each other – but their everyday world is one of processing DNA samples according to established protocols. Thus, when peritos tried to explain why La Tabla had four populations, without really knowing the logic behind their creation, they usually pointed to Paredes’ office, if we were at the INMLyCF, or referenced the debate between Paredes and Diogenes, which is a shorthand way to synthesize what they considered to be population geneticists’ theoretical disputes. In that sense, while being producers of ‘truth’ (about DNA identification), they are consumers of the ‘truths’ produced by population geneticists.

For the peritos, La Tabla had an ambiguous status and they showed contradictory tendencies in relation to it. They tended to downplay genetic and especially racial difference. We saw this manifest in two ways. First, in the peritos’ everyday experience, no matter which of the four reference populations was used, a similar LR (or random match probability) and/or paternity index was obtained:

If you do paternity tests with any genetic region, regardless of where the person comes from, you will see that the paternity index does not change you will still have 99.999%, and the LRs are also very similar … so it shows that we don’t have great genetic diversity here in Colombia. (Perito 1, Fiscalía)

This clearly undermines the premise that Colombia can be easily divided up into different regions with particular racial-genetic characteristics through La Tabla’s thirteen STRs. However, as discussed below, the claims of population geneticists, such as Yunis, that genetic regions can be differentiated using other markers such as single nucleotide polymorphisms (SNPs) resonates with many peritos.

Second, our focus groups and interviews found that ‘race’ was an uncomfortable word for peritos and forensic experts in the INMLyCF and in the Fiscalía. Occasionally explicit mentions of race – or race-like differences – arose when talking about the logics of making populations or evaluating distinctions. For example, during one of the focus groups a forensic specialist said, ‘you see, you made me say race … it is just that this word has been used as a justification for terrible things in the past … we know there are no pure races’ (Focus group, INMLyCF, 31 January 2012). We found in these contexts that talking about race was understood as talking about purity; therefore, the discussion is not about whether race is a valid biological category, nor if ‘populations’ are intertwined with racial categories; instead the argument is that racial admixture has been so thorough in Colombia that no claims of race (i.e. purity) can be made. (This was also a common theme in the public focus groups we conducted in Bogotá and Medellín; see Schwartz-Marín and Wade, 2015.) In a focus group at the INMLyCF (18 February 2012), participants made reference to the way in which violence and human displacement have accelerated *mestizaje* in the already very mixed landscape of Colombia:

The two great migrations: the first one between 1948 and 1950, called the time of violence, marked by the fight between the Liberals and the Conservatives for political power, well, that made many people move from their original places; on top of the cultural, ancestral admixture, add this layer of displacement. Then, add the forced displacement that derived in the second huge migration, a product of the conflict of the 80s and 90s: this makes Colombia the *mare magnum* of genetic composition. And then of course, there is no structure that you can call ‘pure’, more or less ‘pure’, simply because there is none … people move from one place to another and then, of course, they mix.

The way ideas of race (as purity) were submerged into ideas about a generalized mixture was reinforced by the peritos’ constant reiteration that forensic genetic identifications are based on genetic markers that they understand as ‘bioethical’. The markers are seen to avoid possible ethical problems related to racial, and potentially racist, identifications because the STRs used for the forensic work are located in non-codifying regions of the genome and are thus seen to be unrelated to race or physical appearance.^8^ The mere mention of race often evoked the possibility of racism and discrimination, which could be avoided by the use of markers seen as ethically appropriate (field notes, February 2011; see also Abu El-Haj, 2012: 23).

It is clear that other tendencies were also at work among the peritos, contradicting the denial of regional and racial diversity and instead reinforcing the basic premise of Colombia as regionally and racially differentiated. Importantly, these processes did not generally involve direct references to race, but instead used the language of region, with racial difference figuring as a subtext. The imaginaries of race, region and the composition of the Colombian nation are built into the forensic process from the very start, beginning with the way a case is filed and opened. As soon as a case receives a criminal file or number, it is assigned to a department in the Colombian territory and then to one of the four different populations defined in La Tabla. Material evidence will be checked to see if there are useful biological traces, and if found, it will then be sent to the genetics laboratory to be processed. Once it is determined that there is enough forensic evidence (DNA) to run a polymerase chain reaction (PCR) and an identification kit, genetic analysis will start. In the final report produced by a perito for a judicial case, the populations are reiterated. Such a report might say, ‘The Andean Region of Colombia was the reference population, and its frequencies have been previously analysed and reported by the laboratory of genetics at Instituto Nacional de Medicina Legal y Ciencias Forenses’ (meaning the publication by Paredes et al., 2003).

When we asked how reference populations were assigned to bodies, suspects or potential parents, the responses we got from peritos at the INMLyCF were always the same: It was an assumption based solely on the location of the crime scene, mass grave or parental dispute. No other criteria could be used by the peritos since they only dealt with the biological samples and had no access to any other case information, such as pictures or case narratives (peritos argued this allowed them to remain objective). Therefore, no criteria based on appearance or self-declared ancestry could be used to assign a reference population to a case. We found these bio-geographical assumptions troubling, given that close to 5 million people have been internally displaced in Colombia. When we asked the peritos about internal displacement and migration, they agreed that such assumptions could be misleading, but still necessary:

Yes, I know. For example, it could happen that both parents come from the Chocó (north Pacific coast), and they met in Bogotá, and they filed the dispute here in the city, consequently we will be using the Andean population as the referent … despite the fact they are not Bogotanos. But I still need a reference population to calculate the LRs. (Paraphrasis, fieldnotes, 30 March 2012)

The public imaginary of Colombia as regionally and racially differentiated was part of the common sense of the peritos as members of the Colombian nation. In a focus group at the INMLyCF (18 February 2012), many peritos and forensic specialists recognized that Colombia is a very mixed nation, but other members of the focus group insisted that, despite this mixture, clear distinctions between different regions in Colombia and the people living in them could be made:

The regionalization of our country is born of the natural regions; at least that is what I think, right? One thing is the environment in the high mountains … natural regions delimited by the height of the mountains basically, or the coasts, which makes contrast with the sea, the interior of the country, the mountain, you know … so I think that a person who lives at sea level behaves in a very different way. I would not reduce it to only that, but I think it has a lot to do with it.

In this account, words such as ethnicity and/or race are avoided, and the weight of regionalization is given to geography and climate. But these ‘natural regions’ also were immediately articulated by other members of the group in terms of people living within them, citing the idea that ‘the south of Colombia is visibly more indigenous’ or that certain highland areas are more European, being home to groups like the *paisas* (the people of the Antioquia region), reputed to be relatively White in Colombian terms. When talking with the peritos at the Fiscalía, they resorted to a similar regional–racial repertoire to explain difference in Colombia. Although they agreed on the idea that in Colombia ‘*somos muy mezclados*’ (we are very mixed), the notion that it made sense to divide the country into regions was strongly articulated as well: ‘That is what they teach us from our early days at school’.

The following excerpt of a conversation with J.E., a molecular biologist who trained at the INMLyCF and introduced us to the field of forensic genetics there, shows the deeply rooted image of Colombia as regionally and racially differentiated and demonstrates the way in which common sense and the ‘visible’ differences among Colombians work in the stabilization of scientific knowledge:^9^

J.E.: I think Colombia is one of the most mixed countries there is … here we are a *mescolanza* (a mixture) – of an almost weird kind – because a bit of everything came to the country: black slaves, our indigenous people, lots of Europeans and here everyone mixed with everyone.

E.S.M.: That is precisely why I am so interested in why you divide the country in regions, when there is no significant genetic distance.

J.E.: Maybe the Afrodescendants. Why? Because that is a region where most of the people are black, and of course there is a great difference at least phenotypically, of course there is. [… And] in the south of the country nowadays are the people who most conserve their indigenous structure, their social ways; the *cabildos* [indigenous councils] still exist, their indigenous reservations still exist … they still keep a lot of that, there is also certain degree of endogamy, it is effectively a closed population. Not a lot of people enter or leave the community, that is the reason for saying that they are pure indigenous, right? … Then, in the Caribbean region many Jewish people arrived afterwards, many Muslims, many Arab people, which might have been the reason to treat it as an isolated region. […] For instance if we were looking at the *paisas*, if we were to look into a deeper level in Antioquia, we might find these [genetic] differences and the same thing could happen in other regions, because of endogamy. The same happens in the Atlantic Coast, in Palenque de San Basilio [previously a maroon community], where the slaves who were able to escape made their own town and stayed there; those who live there now are the great-great-grandsons of the original slaves. Then I would say that there is a substructure, especially one that tends to be African, they are descendants of real slaves, who were brought from Africa against their will … So again I think it all depends if your magnifying glass allows you to see what is very, very small. For me the way we are looking at it does not allow us to see that …

In their scientific and practical context, peritos mobilize common-sense ideas about the diversity of the Colombian nation including the demographic history taught in school and obvious phenotypic differences such as skin colour to reinforce the idea that Colombia is made up of populations-regions, despite the challenges to such a concept presented by the facts of the mestizaje, violence-related displacements and the lack of genetic diversity. J.E. believes that the degree of resolution limits the scientific gaze, while not considering other explanations, such as the possibility that the regionalized cartography of Colombia does not have a genetic counterpart. During the focus group at the Fiscalía, a similar repertoire of explanations – which links the phenotypically ‘obvious’ to the molecular – was given to us when explaining the fact that genetic distances do not justify the categories made by Paredes et al. (2003). The region emerged as an obvious criterion for classification. As one perito said, ‘here we separate it [DNA] by regions, Colombia by regions, for us it is easier to separate them [the samples] by regions’:

E.S.M.: So why divide the country in regions, if there aren’t significant differences?

Perito 1: Well, that is a good question [generalized laughter].

Perito 2: […] Definitely you do need to do a kind of division, at least demographically, of the Colombian population … we are definitely not the same, I mean if you travel to the Pacific coast of Colombia you will find a totally different race, from what you find here in the centre of the country, and the same will happen if you go to the eastern side of our country, you will find lots of indigenous population, the same in the southwest, and they are not the same … we are not the same …

In sum, despite their objections to the idea of race and their commitment to using what they see as ‘bioethical markers’ and despite their recognition that the regional–racial populations of La Tabla made no statistical difference in producing LRs, La Tabla and its categories continued to work smoothly in the context of the peritos’ laboratories and work protocols. This is because regions were a common-sense reality for these practitioners and because La Tabla could still be used to produce valid results for legal purposes.

This is an example of public science, as we use the term in this article, in two senses: (a) insofar as public imaginations of Colombia as a country with distinctive racialized regions facilitated the routine use and normalization of La Tabla and (b) insofar as the peritos’ concern with being seen to endorse ideas of race (as purity) and to potentially be involved in distinctions that smacked of racism – both morally inimical to the peritos’ imagination of the Colombian public sphere – pushed them to avoid explicit mention of race and accept the fact that, in practice, La Tabla’s four populations produced very similar LRs.

In the next section, we argue that, in a different context – that of the courtroom – the regional–racial populations produced by La Tabla are once more erased or at least back-grounded and overshadowed by the apparent power of forensics to make secure identifications, irrespective of region or race.

## Forensic genetics in the courtroom

Normally, before public hearings dealing with genetic data take place, a report is produced by peritos to communicate the LRs of DNA profiles or paternity indexes to their legal audiences. It is through this document that peritos provide their expert evidence. It is designed to be circulated to public prosecutors or other actors in the public sphere and its contents detail the laboratory processes and scientific standards that led to the forensic statement. In Colombian forensic practice, LRs can be as high as 21,265 trillion:

From the previous calculation it can be said that the genetic profile we analysed is found in one of 21,265 trillion persons in the reference population used for this case. This result means that it is 21,265 trillion times more probable that the evidence analysed comes from [the suspect, the putative relative] than from another random individual in the reference population. (Quoted from the institutional documentation used to communicate forensic genetics results obtained at the INMLyCF)^10^

The courtrooms are places in which the forensic genetics and the astronomical LRs it yields are taken to be responsible for ‘80 or 90 percent’ of the weight of evidence (focus group with public prosecutors, 2012). Throughout the public trials that we witnessed during our fieldwork, deference towards genetics was the norm and it was common practice to ask genetic specialists to translate their knowledge into everyday language: ‘*explíquenoslo en cristiano*’ (explain it to us in Christian language). Public prosecutors and attorneys have told us that, although in Colombia there is no official hierarchy of evidence, ‘DNA is what the judges and victims are looking for, and we feel confident with our cases when we have it … defenders know this, and they do whatever they can to avoid their clients’ getting a genetic test’. Public prosecutors told us that as part of their professional routine and legal practice when dealing with genetic profiles, they like to ask the genetic specialists the following question when presenting their case in a public audience or hearing:

Prosecutor: Please tell us how many human beings living today on planet earth we would need to find the same genetic profile by sheer chance?

Perito: According to our reference population, we would need approximately 3 times the population of planet earth to find the same genetic profile by sheer chance.

LRs linked to DNA profiles are so high that what is commonly known as ‘genetic proof’ among legal specialists becomes certainty in everyday juridical practice (cf. Kahn, 2012). Despite their central role in the construction of ‘certainty’, populations and the work they do mainly go unnoticed and unquestioned in the courtrooms. Different from what peritos think happens in the European and US courtrooms – where they assume that the validity of DNA matching is sometimes called into question (Lynch et al., 2008; M’charek, 2000, 2008) – none of the attorneys, forensic specialists and policemen we interviewed during our fieldwork could remember any instance in which this had happened in Colombia’s newly established accusatorial system. On the contrary, to paraphrase the attorneys we talked with, ‘you might not lose a case if you don’t have DNA, but you will definitely win it if you do’.

In the expert reports produced by peritos and circulated to defenders and public prosecutors, the idea that Colombia is composed of genetic regions is routinely reproduced. As we saw above, such reports name the reference population used to calculate the LR – for example, ‘The Andean Region of Colombia was the reference population’. However, interestingly, most of the prosecutors and government attorneys we interviewed did not know about the use of reference populations to produce genetic profiles. During our focus groups, genetic profiles and fingerprints were described as the two main identification techniques. The two attorneys and police investigators who were aware that populations were used to produce genetic profiles assured us (after consulting with each other) that the DNA of individuals was compared against a global referent: the regionalized genetic populations of La Tabla were obscured, despite being written in every forensic report as a part of the standardized protocol.^11^ We found that, when preparing for a trial, prosecutors and defence lawyers had never considered the possibility of contesting DNA evidence in terms of the adequacy of the reference population being used by the peritos. As one prosecutor told us, hesitantly,

Yeah, yeah, I know the probabilities are based on the different regions in Colombia, but in my experience I have never questioned the use of the different regions in a genetic test. Nor has the defence attorney […] I mean, what I see is not if the results consider the Andean region, but rather the general Colombian population […] so it is not determined by regions […] The peritos give me the probabilities in terms of trillions, so I just ask: ‘This number equals how many times the human population on earth?’ And they tell me: seven or eight. And then, that’s it. (Interview by Schwartz-Marín and Cruz-Santiago, 6 March 2012)

The public properties of forensic DNA as a ‘silent witness’ (M’charek, 2008), capable of delivering truth despite the effects of extreme violence that, for example, hides dismembered body parts in different locations, is well established in Colombia’s courtrooms, among its police forces and among the many organized victims we interviewed. When we interviewed the judge of a sexual assault case, in which two DNA samples taken from the suspect had given contradictory results – for reasons unknown – he continued to insist on the irrefutable ability of DNA tests to reveal the identity of criminals and victims; this was despite the fact he was unfamiliar with most of the technicalities of forensic genetics such as the existence of reference populations or the existence of the database in which genetic profiles were stored. For him, DNA was a unique barcode through which one DNA profile (crime scene sample) was simply matched to another (suspect criminal or victim). However, DNA’s silent witness status is an achievement rather than a natural property of forensic practice. The disappearance of racial, ethnic or national referents in genetic profiling in Colombian courtrooms is not only derived from the idea that DNA is a unique barcode but, as we have already mentioned, is also linked to the insistence by peritos that forensic genetics uses ‘bioethical markers’ that are not linked to race.

Narratives that emphasize the power of DNA commonly construe it as the ultimate individualized proof of identity in the public sphere. In Colombian courts, this dominant ‘silent witness’ narrative overwhelms the regional–racial populations that are used by the peritos to produce the LR or random match probabilities that prosecutors deploy and judges consume. The public enactment of genetic fingerprinting or profiling makes it possible to establish a strong statistical link between individuals and biological material while at the same time sidestepping potentially troubling racialized narratives. Indeed the conceptual and practical separation made between race, as a visible character of bodies, and DNA, as an invisible yet essential property of the same bodies, makes forensic genetics a powerful tool for individual identification that transcends racial or ethnic difference not only for forensic experts but also in the public courtrooms and in the media reports of courtroom forensic DNA identifications, which generally background racial difference and present the matching process as neutral and heroic (Díaz del Castillo et al., 2012).

## Conclusion

By approaching forensic genetics in Colombia as a form of public science, we can understand the successful stabilization of DNA populations and the re-articulation of race and region in forensic genetic practice. In the courtroom and among the criminal investigators, public prosecutors, and relatives of the disappeared, the regionalized-racialized populations of Colombian forensic genetics tend not to be seen, leaving instead only the typical astronomical probabilities of DNA profiling. Genetic profiling assumes proof of identity that transcends race and appearance; it travels as a set of ‘bioethical markers’ unrelated to the physical appearance of a suspect or victim. Thus, genetic profiling does not lie, does not forget and, even better, because it is apparently blind with respect to racial difference, it is incapable of discriminating. The dominance of the ‘silent witness’ narrative helps to obscure the racialized dimensions intertwined with forensic genetic practice and constructs forensic genetics as an individualized and unique barcoding technique legitimated by its powerful statistical apparatus.

The disappearance of racial difference in the courtroom is also due to the way forensics figures as pivotal to national efforts to find justice and reparation in the context of widespread civil violence. Ironically, the emergence of La Tabla was linked, as we showed above, to efforts to improve public confidence in the reliability of state forensics. But the regional populations La Tabla created to enhance the accuracy of identification become unimportant in the context of a search for justice which is seen as, ideally, surpassing and over-riding divisions of region and race in a search for national unity.

Following La Tabla from the world of population geneticists to the everyday practice of peritos allowed us to bring forth the workings of common sense, in terms of how La Tabla operates in the standardization of science and as it fuels disputes about the categories it underwrites. For population geneticists, La Tabla’s forensic genetic reiteration of the common-sense notion that Colombia is a country of regions, inhabited by different populations, often described in racial terms, is both redundant (it merely states the obvious) and problematic in genetic terms (the specific techniques used in forensic genetics are not adequate for a proper population genetics). However, many population geneticists also reproduce the standard images of Colombia differentiated by racialized regions, and it is precisely because of the same common-sense constructs that peritos do not mind glossing over the lack of genetic distances between regional populations in La Tabla.

Drawing on common-sense historical and demographical knowledge of the Colombian nation, as well as phenotype differences of ‘others’ deemed to be Black in the coastal regions, indigenous in the south and more European in the highlands, peritos link the visible and apparent to the molecular. Here, common sense predates and supports the categories created by scientific knowledge; science will eventually confirm what everyone already knows about the nation and its ethno-racially endogamous or mixed landscapes. Questions about the proper place of racial categories and their relation to nature and ‘reality’ do not bother forensic specialists in the same way as they bother those devoted to population genetics. The way in which racial categories matter or do not is one of the boundaries constructed by population geneticists to differentiate themselves from their practice-oriented forensic counterparts, the peritos.

Strategies that draw on both history and appearance are a common tactic in lay engagement with genetic science in Colombia (see Schwartz-Marín and Wade, 2015). With respect to population genetics, we have shown that the forensic laboratory is another space in which we can think of interactions between public domains and genetic science (Lévy-LeBlond, 1992). Common-sense imaginaries shape how peritos use and understand La Tabla: explanations linking the visible and the invisible observed among the peritos are strikingly similar to those found outside the confines of forensic expertise.

In their everyday practice, peritos are selectively crafting many knowledge registers at the same time: what is seen by population geneticists as inconsistency and the imposition of pre-conceived frames in La Tabla are, for the peritos, questions of scale of resolution and representative sampling. In addition, race and its relation to genetics is less problematic for the peritos who tend to deploy differences of physical appearance to make sense of the genetic and regional composition of the Colombian nation. At the same time, making connections between racial appearance and genetics is seen as potentially discriminatory, a problem that can be circumvented by referring to the bioethical status of forensic genetic markers that are disconnected from phenotype.

In sum, the relations between race and nation in forensic science need to be constantly managed, and despite all the statistical and mathematical tools devised to produce certainty, common sense and dominant imaginaries of difference are still fundamental to the way population geneticists and peritos conceptualize and dwell in their life-worlds, shaping the assumptions that delimit genetic populations and the symbolic frames they use to engage and produce further knowledge with them. What is striking about the Colombian material explored here is the power of the racial–regional imaginary to inflect, in varied ways, the world of forensic genetics – its researchers and technicians – and population genetics more widely. In Mexico, as noted above, a sense of regional difference has not shaped forensic genetic practice in the same way. This Colombian imaginary is long standing and has been co-produced in the interactions of scientists of various kinds, administrators, politicians, educators and lay people over a long period. Genomics is one more domain where we can detect its presence, as well as attempts, by such as Diogenes, to challenge some of its contours.

Genetics enacts tension as it produces representations of racialized regions, which can easily reinforce and naturalize a sense of difference, while simultaneously highlighting the processes of movement, migration and mixture, which are also said to constitute the nation and characterize major cities such as Bogotá. In La Tabla itself, these latter processes remain hidden, as they tend to in most representations of racialized regions, but the peritos clearly emphasized them in their discourse, while in the courtroom regional difference was erased more radically still by individualized identifications assumed to take place in relation to a global population. In that sense, genetics provides a specific language for talking about perennial issues of sameness and difference, without providing a means to resolve the tension between them.

## Notes

1. A short tandem repeat (STR) is segment of the DNA chain where short sequences of DNA are repeated.

2. The ‘racial ancestor’ is inferred from an expert reading of the features of the skulls and is classified using three main parental groups: ‘Caucasoid’, ‘Mongoloid’ and ‘Negroid’. In Colombian practice, almost all skulls are labelled ‘Mestizo’. Those that, according to the forensic anthropologists, are strongly characteristic of one of the three parental groups will be labelled as ‘Caucasoid-Mestizo’, ‘Mongoloid-Mestizo’ or ‘Negroid-Mestizo’.

3. The use of a national referent population, and a systematic and integrated system of DNA analysis, is considered one of the principal virtues of the Colombian forensic system, when compared to other systems in Mexico, Guatemala or Argentina.

4. The number of criminal cases examined each year is about 1500. Approximately 21,000 paternity cases are processed by the Instituto Nacional de Medicina Legal y Ciencias Forenses (INMLyCF) each year, paid for by the Colombian Institute of Family Welfare.

5. There have been public clashes between Paredes and Diogenes regarding La Tabla and the methods, design and logic that led to its creation and publication. Their rivalry is well known.

6. Diogenes maintains that in some areas of the country a relation can be established between the region and the genetic structure of its inhabitants, as in the indigenous Wayúu region. In the case of other places, some of which were even thought of as genetic isolates, like the Caribbean island of San Andrés, mostly populated by African descendants, no clear or significant genetic differences are found with the rest of the country.

7. Diogenes has been tackling the problem of using different categories than those commonly used and endorsed by population geneticists to describe human diversity. This has been done with varying degrees of success, as well as pitfalls; for an account of such endeavours, see Olarte Sierra and Díaz del Castillo (2014).

8. Peritos acknowledged that avoiding race was difficult because the forensic reports circulating in the courtrooms might describe populations with terms such as ‘African’, ‘Amerindian’ or ‘European’, which sometimes elicited questions from prosecutors about whether the genetic test could determine the race of the suspect or victim.

9. Taken from field notes and recording, by Schwartz-Marín and Cruz-Santiago, 9 May 2012.

10. The reference population – considering Colombia as a whole – would be roughly 40 million; however, likelihood ratios (LRs) would be 21,265 trillion. This is because LRs are calculated on the possible combinations of STRs, not individuals.

11. Most of our interviewees had been working in the INMLyCF for more than 16 years and in that time not one of them had any experience of an attorney, public prosecutor or defendant who made explicit mention of the four reference populations used in genetic profiling. Likewise, none of our interviewees working as lawyers in sex crime units or the units for disappeared persons had any idea about the use of reference populations. We also followed the trial of Néstor Romero Paipa from 2011 to 2012, charged with sexual abuse, and viewed dozens of trial reports and several trial videos in which DNA evidence was crucial to the case. Although in all of them the reference population is mentioned, as required by law, such mentions did not occur in the arguments happening in the trials, where instead the world population was used as the yardstick to evaluate the veracity of DNA evidence.
